# Effect of Algorithmic Music Listening on Cardiac Autonomic Nervous System Activity: An Exploratory, Randomized Crossover Study

**DOI:** 10.3390/jcm11195738

**Published:** 2022-09-28

**Authors:** Alfredo Raglio, Roberto Maestri, Elena Robbi, Antonia Pierobon, Maria Teresa La Rovere, Gian Domenico Pinna

**Affiliations:** 1 Music Therapy Research Laboratory, Istituti Clinici Scientifici Maugeri IRCCS, 27100 Pavia, Italy; 2 Laboratory for the Study of Ventilatory Instability, Department of Biomedical Engineering, Istituti Clinici Scientifici Maugeri IRCCS, 27040 Montescano, Italy; 3 Laboratory for the Study of the Autonomic Nervous System and Cardiorespiratory Activity, Department of Cardiology, Istituti Clinici Scientifici Maugeri IRCCS, 27040 Montescano, Italy; 4 Psychology Unit, Istituti Clinici Scientifici Maugeri IRCCS, 27040 Montescano, Italy

**Keywords:** cardiac autonomic nervous system, physiological parameters, music therapy, music listening, algorithmic music

## Abstract

It is proven that music listening can have a therapeutic impact in many clinical fields. However, to assume a curative value, musical stimuli should have a therapeutic logic. This study aimed at assessing short-term effects of algorithmic music on cardiac autonomic nervous system activity. Twenty-two healthy subjects underwent a crossover study including random listening to relaxing and activating algorithmic music. Electrocardiogram (ECG) and non-invasive arterial blood pressure were continuously recorded and were later analyzed to measure Heart Rate (HR) mean, HR variability and baroreflex sensitivity (BRS). Statistical analysis was performed using a general linear model, testing for carryover, period and treatment effects. Relaxing tracks decreased HR and increased root mean square of successive squared differences of normal-to-normal (NN) intervals, proportion of interval differences of successive NN intervals greater than 50 ms, low-frequency (LF) and high-frequency (HF) power and BRS. Activating tracks caused almost no change or an opposite effect in the same variables. The difference between the effects of the two stimuli was statistically significant in all these variables. No difference was found in the standard deviation of normal-to-normal RR intervals, LF_power_ in normalized units and LF_power_/HF_power_ variables. The study suggests that algorithmic relaxing music increases cardiac vagal modulation and tone. These results open interesting perspectives in various clinical areas.

## 1. Introduction

A large body of research has documented how different musical approaches can have a strong therapeutic impact in many clinical fields [1]. However, the validity of reported results in relation to different types of musical stimuli deserves scrutiny.

Neurophysiological and neurochemical effects of active and receptive music approaches are well supported by several studies [2,3,4,5], including the effects on cardiorespiratory parameters like heart rate (HR), respiratory rate (RR) and heart rate variability (HRV) [6,7,8,9,10,11]. However, the results are frequently inconsistent [8] and the relationship between music stimuli and their effects is still unknown. The need to better understand this relationship derives from the general complexity and heterogeneity of music stimuli, as well as from methodological weaknesses of the studies in terms of design, sample size and, especially, unclear music stimuli definition and treatment (self- or experimenter-selected music, rationale and administration criteria, music features description, setting, delivery schedule, etc.) [12].

This study investigates the impact of two clearly-defined, experimenter-selected music stimuli, one activating and another relaxing, on a set relevant non-invasive markers of cardiac autonomic nervous system activity using a sound experimental design and in-house developed signal analysis algorithms [13,14,15,16,17,18]. We hypothesized that the two music stimuli would have a different impact on cardiac autonomic activity and that this difference would be consistent with their supposed effect (e.g., a reduction in HR during the relaxing stimulus and an increase during the activating stimulus). 

The important innovation of this study concerns the use of algorithmic music (Melomics-Health) created with specific therapeutic aims [19]. The use of Melomics-Health music allows to create standardized and homogeneous stimuli bypassing cultural elements and the complexity of conventional music. 

## 2. Materials and Methods

### 2.1. Subjects 

Subjects for the study were 22 healthy volunteers, 9 men and 13 women, aged 34.5 ± 12 years (range: 21–58). None of the subjects practiced intensive sports activities or took drugs potentially interfering with cardiac autonomic activity. Baseline systolic and diastolic arterial pressure were 118.5 ± 13.7 mmHg and 74.8 ± 10.5 mmHg, respectively. The study was approved by the Ethical Board of the Istituti Clinici Scientifici Maugeri, Pavia, Italy (2420 CE, 23 April 2020) and all subjects provided written consent to the participation in the study.

### 2.2. Music Intervention

The neuroscientific literature bases the effectiveness of music on its predictability and familiarity for the listener. Indeed, our perceptual system can predict and recognize musical patterns, and our reward system is satisfied by the emotional recognition of such patterns [20]. However, we cannot exclude that some effects (in the case of this study physiological effects) may result from the subjective pleasure of listening but also from specific music structures and specific music parameters [21,22].

**Figure 1:**
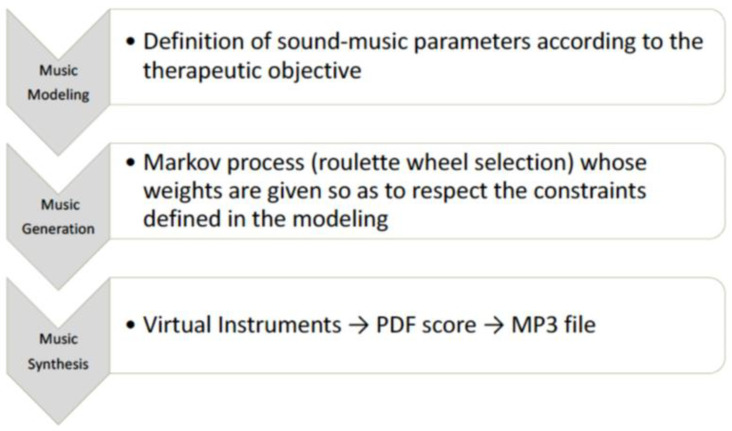
Algorithmic Music (creation process).

The music used in the study was composed by the Melomics-Health algorithm [19,21] (Figure 1) that composes music tracks (melodies whose notes belong to the temperate system) based on specific sound parameters and appropriate musical structures to achieve the expected therapeutic objective. This way of composing music gives the possibility to work on the design of the music itself, thereby creating the necessary conditions for its use in the therapeutic field. In fact, one of the advantages of this technology is the possibility of shaping and modelling music according to the therapeutic objective. The algorithm has already been used in some experimentations in the field of pediatric acute pain, stress, and radiotherapy [23,24,25]. For this study, Melomics-Health created two couples of pieces for cello (couple 1) and clarinet (couple 2), lasting 5 min each. Each couple consisted of one relaxing and one activating track. Short examples from tracks are reported in Supplementary Materials (audio samples 1, 2, 3, 4).

### 2.3. Experimental Protocol

**Figure 2:**
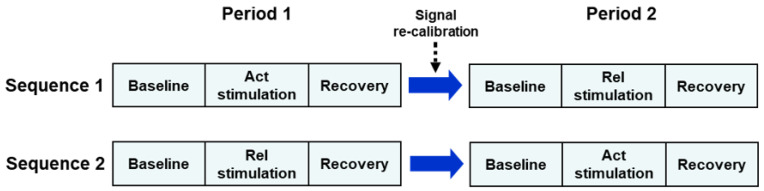
Schematic diagram of the experimental design of the study. Each recording subsection (baseline, stimulation, recovery) lasted 5 min. Act = activating; Rel = relaxing.

The study was conducted using a two-period, two-sequence crossover design comparing the activating music model (Act) with the relaxing model (Rel). Subjects were randomly and evenly (1:1 ratio) allocated to the Act→Rel or Rel→Act sequence. Subjects in the Act→Rel sequence (Sequence 1) received the activating music stimulus during the first recording session (Period 1) and, after signal re-calibration, received the relaxing stimulus during the second recording session (Period 2) (Figure 2). Subjects in the Rel→Act sequence (Sequence 2) received the Rel stimulus during Period 1 and the Act stimulus during Period 2. Each session included a baseline (5 min), a stimulation (Act or Rel, 5 min) and a recovery sub-section (5 min) (Figure 2).

Recordings were carried out in the sitting position on a comfortable armchair, in our laboratory for the Study of the Autonomic Nervous System and Cardiorespiratory Activity. After instrumentation and signal stabilization, we recorded the ECG, lung volume through respiratory inductive plethysmography (Q-RIP Respiratory Effort System, Braebon Medical Corporation, Kanata, Ontario, Canada) and continuous non-invasive arterial blood pressure (CNAP, CNSystems Medizintechnik GmbH, Graz, Austria). 

### 2.4. Signal Analysis and Measurement of Autonomic Indices

The ECG and arterial blood pressure signals were processed by dedicated software [13] to obtain beat-to-beat RR interval and systolic arterial pressure (SAP) time series. Both time series were visually inspected simultaneously and the widest segment free from artifacts, large transients or marked changes in the oscillatory pattern of the signals, was interactively selected [26]. After correction of isolated ectopic beats by linear interpolation, mean heart rate (HR), the standard deviation of normal-to-normal RR intervals (SDNN), the root mean square of successive squared differences (RMSSD) of NN intervals, and the proportion of interval differences of successive NN intervals greater than 50 ms (pNN50), were computed [27]. 

In order to compute spectral indexes of cardiovascular variability, a 2-Hz re-sampled version of RR and SAP time series was obtained by cubic spline interpolation. After detrending via least-square second-order polynomial fitting, the power spectral density of the RR time series in the low-frequency (LF, 0.04–0.15 Hz) and high-frequency (HF, 0.15–0.45 Hz) bands was estimated by the autoregressive method (Burg algorithm) with spectral decomposition (Johnsen and Andersen algorithm). The autoregressive model order was set at 26, but was interactively increased when negative components appeared in the spectral decomposition table [26]. In order to cope with the possible presence of more than one spectral component within each band, we computed the power of the LF and HF bands as the sum of the powers of the spectral components identified in each of them [17]. Components showing < 10% of the overall power in the band were ignored as they probably represented pure noise contributions. The LF power in normalized units (LFnu) was computed as: LF power/(LF power + HF power)*100. The HF power in normalized units (=100 − LFnu) was not analyzed, to avoid redundancy in the results.

Baroreflex sensitivity (BRS) was estimated by computing the average value of the transfer function modulus between SAP and RR interval time series in the LF band [14]. 

### 2.5. Statistical Analysis 

The effect of each music stimulus on a given autonomic index was calculated as the difference between the value of the index during stimulation and that during the preceding baseline condition. The analysis was performed using a two-factor analysis of variance: Sequence (two levels: Act→Rel, Rel→Act) and Period (two levels: 1, 2), with repeated measures on the factor Period. The test for the Seq factor is a test for the presence of a carryover effect, which is a potential drawback of any crossover design, whereas the test for the Period factor is a test for the presence of a systematic drift in autonomic indexes between the first and the second part of the experiment. We hypothesized that such a drift could occur due to the length of the recording (about 35 min) and the consequent possibility of developing drowsiness or irritation over time. Finally, the test for the interaction between the factor Seq and the factor Period is a test for the difference between the effects of the two treatments (i.e., effect of Rel stimulation vs effect of Act stimulation; null hypothesis: effect of Rel stimulation = effect of Act stimulation). Before analysis, the normality of the data was checked by the Shapiro–Wilks test, and an attempt was made to convert non-normally distributed variables into normally distributed variables using variable transformation. Moreover, treatment effects were analyzed in these variables using a non-parametric approach (Wilcoxon test). One-sample tests were performed by the t-test or Wilcoxon signed-rank test, when appropriate. All statistical tests were two-tailed and statistical significance was set at *p* < 0.05. The analyses were carried out using the SAS/STAT statistical package, release 9.4 (SAS Institute Inc., Cary, NC, USA).

## 3. Results

Summary statistics (mean ± SEM) of mean heart rate and cardiovascular variability indices in the two baseline conditions and during the subsequent relaxing and activating musical stimulation are shown in Table 1. Summary statistics of the difference between the value of these variables during stimulation and that during the preceding baseline condition (stimulation effect), as well as the significance probability of the tests for the carryover and period effects, and for the difference between the effects of the two treatments, are shown in Table 2. For the sake of completeness of information, the same tables are also reported in the Supplementary Materials (Tables S1 and S2) using a different descriptor of the central tendency and dispersion (median (Q1, Q3)). In some variables (RMSSD, LF power, HF power, LF/HF), the distribution of the effect was non-normal in a subset of the four measurement conditions, therefore it was not possible to find a variable transformation capable at the same time to achieve normality in some conditions and preserving it in the others. Accordingly, in these variables, testing for the treatment effect was also performed using a non-parametric approach. 

The relaxing stimulus caused a decrease in HR and an increase in RMSSD, pNN50, LF power, HF power and BRS, while the activating stimulus caused almost no change or an opposite effect in the same variables. The difference in effect between the two stimuli was statistically significant in all these variables (last column of Table 1). No difference in effect was found in the SDNN, LFNU and LF/HF variables (*p* > 0.30 for all).

The change in breathing frequency was −0.005 ± 0.36 Hz during the relaxing stimulus and 0.005 ± 0.024 Hz during the activating stimulus. The difference between these effects was largely non-significant (*p* = 0.24).

## 4. Discussion

This study shows that two different musical stimuli (relaxing and activating) obtained through the use of Melomics-Health, a novel algorithmic music capable of providing standardized stimuli with specific therapeutic aims, have a significantly different impact on cardiac autonomic nervous system activity as assessed by the measurement of mean HR and standard cardiovascular variability indices. 

The study evaluates the short-term effects of algorithmic music. A major aspect of this approach is the possibility of relating structures and music parameters to the effects produced. This is not possible with conventional music pieces where structures are complex and overlapping. Algorithmic music consists of parameters and structures that can be identified, reproduced and modulated in relation to the effects produced. These aspects are of fundamental importance in the field of music therapy. Indeed, the modeling of musical pieces with a clear de-activating rather than activating effect is a particularly important resource in different therapeutic clinical contexts: temporary or structural anxiety, stress, behavioral disorders, etc. This study shows with rigorous and sensitive measurements clear effects on crucial markers of parasympathetic activity. In particular, the decrease in HR with the algorithmic relaxing music model constitutes a result of considerable interest, congruent with the increase in RMSSD, pNN50 and BRS that reached a statistical significance in the evaluation of this effect. 

### 4.1. Interpretation of Changes in Cardiovascular Variability Indices

Based on the classical Rosenblueth–Simeone model [28], the decrease in mean HR observed during the relaxing musical stimulus can be interpreted as the effect of an increase in tonic vagal activity, a decrease in tonic sympathetic activity or both. The RMSSD, pNN50 and HF power are well-known correlated markers of cardiac vagal modulation [29,30]. These indices increased during the relaxing stimulus and showed an opposite trend during the activating stimulus, thus indicating that vagal modulation was enhanced by the former and possibly lessened or left almost unchanged by the latter. 

Several studies have demonstrated the capability of BRS, as estimated non-invasively by the transfer function method, to detect the change in cardiac baroreflex function following structural cardiovascular disease [14], as well as its clinical and prognostic relevance [16,18,31,32]. Using a beat-to-beat mathematical model of baroreflex blood pressure and HR control and simulating different physiological and pathological hemodynamic and autonomic conditions, van de Vooren et al. showed that BRS is almost exclusively vagally mediated and that, accordingly, the LF oscillations of HR mainly represents vagal transmission of the corresponding blood pressure oscillation via the baroreflex [33,34]. The latter finding is consistent with a number of investigations on healthy human subjects using a similar or different methodology to estimate BRS [35,36,37,38]. In our study, we found that BRS and the LF power had significantly opposite changes during the two musical stimulations, namely an increase during the relaxing stimulus and a decrease during the activating stimulus, suggesting a vagally mediated increase in baroreflex sensitivity during the former and the opposite during the latter. 

The LF/HF and LF_NU_ have long been proposed as two mathematically-related indexes describing the so-called “sympathovagal balance” [39]. This concept, however, has been heavily criticized on the grounds that it lacks a precise definition and a sound physiological construct, and that the response of the LF/HF and LF_NU_ to some autonomic conditions does not reflect the underlying changes in the physiological state [40,41,42,43,44]. This might explain why we found no difference in LF/HF and LF_NU_ between the responses to the two musical stimuli (treatment effect: *p* > 0.60 for both), and why the two responses separately were largely non-significant (*p* > 0.85 for both). 

Finally, the SDNN is a measure of global HR variation, thereby including both sympathetic and vagal modulations. A known limitation of this index is its dependence on the length of the recording period [27]. Indeed, during short-term recordings, as in the present study, the SDNN is markedly influenced by the slow trends resulting from fragments of very-low and ultra-low HR oscillations [27]. These issues make the interpretation of a non-significant treatment effect rather difficult. 

Based on all these considerations, our interpretation of the study findings is that the relaxing stimulus increased cardiac vagal modulation, and, possibly, also cardiac vagal tone, while the activating stimulus had an opposite effect or no effect at all. 

### 4.2. Study Strengths and Limitations

Although several studies have shown that music listening may provide therapeutic effects, such effects can hardly be attributed to a specific type of music [45]. This study is an attempt to assess the effects of a specific type of music (algorithmic music) whose structure and parameters are known in detail. These effects were tested using physiological outcome measures that partially by-pass the subjectivity of music perception, thereby making the assessment process objective. Moreover, previous findings on the impact of music on cardiovascular autonomic parameters were often inconsistent [8], likely because of methodological shortcomings and poor definition of musical stimuli [12]. The present study fills both of these gaps by using appropriately designed and standardized stimuli and a sound methodological approach.

The study was sufficiently powered to detect a significant difference in the outcome of the study in the five parameters in which there was a clear and meaningful trend in descriptive statistics (HR, RMSSD, Pnn50, HF, BRS, Table 1). A largely non-significant result was found in the three parameters in which there was no clear and meaningful trend (SDNN, LF, LF/HF). As for the ancillary analyses shown in the first two columns of Table 2, the lack of adequate power was likely the reason for the non-significance of some univariate tests in which a clear and meaningful trend in descriptive statistics was observed (e.g., HF power).

Two limitations of the study are the limited number of subjects recruited and the fact that only short-term effects on healthy subjects were studied. Another limitation is that the study did not include a control group undergoing conventional music listening (i.e., listening to self-selected music that is subjectively considered to be relaxing or activating). Moreover, in future studies, it might be interesting to integrate the assessment of physiological responses with the evaluation of the subjective impact of music stimuli. Indeed, the latter has already been tested in previous studies [21,23], and could complement the protocol presented in this study. Finally, a potentially critical aspect of the study protocol was the sense of boring often reported by participants and related to the time elapsed from the end of the first stimulation to the beginning of the second stimulation (i.e., recovery, re-calibration and the next baseline). Although, as indicated by the non-significance of the Period effect in all variables (Table 1), this subjective feeling did not translate into a systematic bias in the results, it might have increased measurement variation.

The extent of change in mean HR observed with the relaxing stimuli deserves some comments. Although a one-beat reduction in heart rate might be regarded as of limited physiological value, it is worth saying that several studies have shown that even a one-beat change in heart rate carries relevant clinical value. In particular, the MESA study involving over 5000 asymptomatic individuals without a history of cardiovascular disease showed that for a one bpm increase in resting heart rate there was a 4% greater adjusted risk for incident heart failure [46].

We conclude that the results of this exploratory study are encouraging and open interesting clinical perspectives in various potential areas of application (cardiovascular disease, anxiety and stress conditions, chronic pain, sleep disorders, etc.). To consolidate our findings, adequately-powered randomized controlled trials involving different clinical populations, and including the comparison between algorithmic and traditional music, are needed.

## Figures and Tables

**Table 1:** Summary statistics of heart rate and cardiovascular variability indices in the two baseline conditions and during the subsequent relaxing (Rel) and activating (Act) musical stimulation.

Variable	Rel Baseline	Rel Stimulation	Act Baseline	Act Stimulation
HR, bpm	73.4 ± 2.2	72.2 ± 2.1	73.5 ± 2.1	73.3 ± 2.1
SDNN, ms	42.8 ± 2.6	40.9 ± 2.5	44.7 ± 3.2	40.3 ± 2.8
RMSSD, ms	30.8 ± 2.2	33.0 ± 2.3	35.0 ± 3.4	30.3 ± 2.7
pNN50, %	11.7 ± 2.6	14.1 ± 2.6	12.6 ± 2.7	11.1 ± 2.5
LF_power_, ms^2^	559 ± 87	644 ± 108	712 ± 144	512 ± 105
LF_NUr_, N.U.	51.7 ± 4.4	50.5 ± 4.2	53.9 ± 4.7	53.2 ± 4.1
HF_power_, ms^2^	443 ± 65	519 ± 59	479 ± 90	408 ± 62
LF/HF, A.U.	1.51 ± 0.26	1.37 ± 0.22	1.78 ± 0.34	1.51 ± 0.22
BRS, ms/mm Hg	6.0 ± 0.6	7.0 ± 0.7	6.3 ± 0.6	5.9 ± 0.5

Summary statistics are expressed as mean ± SEM. HR = mean heart rate; SDNN = standard deviation of normal-to-normal RR intervals; RMSSD = root mean square of successive squared differences of NN intervals; pNN50 = proportion of interval differences of successive NN intervals greater than 50 ms; LF_power_ = low frequency power; HF_power_ = high frequency power; LF_NU_ = low frequency power in normalized units; LF/HF = LF_power_/HF_power_; A.U. = arbitrary units; BRS: baroreflex sensitivity.

**Table 2:** Summary statistics of the difference between the value of heart rate and cardiovascular variability indices during stimulation and that during the preceding baseline condition (stimulation effect), with the significance probability of the tests for the carryover and period effects, and for the difference between the effects of the two treatments.

Variable	Rel Effect	Act Effect	*p* Carryover	*p* Period	*p* Treatment
HR, bpm	−1.2 ± 0.4 ††	−0.2 ± 0.3	0.63	0.36	0.018
SDNN, ms	−1.9 ± 1.4	−4.4 ± 1.9 †	1.0	0.82	0.31
RMSSD, ms	2.2 ± 1.0 †	−4.7 ± 1.7 ††	0.87	0.53	0.0002
pNN50, %	2.4 ± 1.1 †	−1.5 ± 0.8	0.95	0.61	0.010
LF_power_, ms^2^	85 ± 50 *	−201 ± 88 *	0.72	0.47	0.015
LF_NU_, N.U.	−1.2 ± 2.7	−0.7 ± 2.6	0.97	0.88	0.89
HF_power_, ms^2^	76 ± 50 *	−72 ± 54	0.69	0.78	0.05
LF/HF, A.U.	−0.14 ± 0.23	−0.27 ± 0.21	0.40	0.85	0.72
BRS, ms/mm Hg	1.0 ± 0.4 †	−0.4 ± 0.4	0.73	0.48	0.018

Summary statistics are expressed as mean ± SEM. HR = mean heart rate; SDNN = standard deviation of normal-to-normal RR intervals; RMSSD= root mean square of successive squared differences of NN intervals; pNN50 = proportion of interval differences of successive NN intervals greater than 50 ms; LF_power_ = low frequency power; HF_power_ = high frequency power; LF_NU_ = low frequency power in normalized units; LF/HF = LF_power_/HF_power_; A.U.= arbitrary units; BRS: baroreflex sensitivity. In some variables with non-normal distribution (RMSSD, LF_power_, HF_power_, LF/HF; see text), treatment effects were estimated using a non-parametric approach. * borderline non-significant (0.05 < *p* < 0.11); † *p* < 0.05; †† *p* < 0.005.

## Data Availability

The data presented in this study are available in the article.

## Supplementary Materials

The following supporting information can be downloaded at: https://www.mdpi.com/article/10.3390/jcm11195738/s1, Audio S1: Excerpt of Cello Relaxing Music; Audio S2: Excerpt of Cello Activating Music; Audio S3: Excerpt of Clarinet Relaxing Music; Audio S4: Excerpt of Clarinet Activating Music. Table S1: Summary statistics of mean heart rate and cardiovascular variability indices in the two baseline conditions and during the subsequent relaxing (Rel) and activating (Act) musical stimulation. Table S2: Summary statistics of the difference between the value of heart rate and cardiovascular variability indices during stimulation and that during the preceding baseline condition (stimulation effect), with the significance probability of the tests for the carryover and period effects, and for the difference between the effects of the two treatments.
