# Supplementary material for: Effect of Algorithmic Music Listening on Cardiac Autonomic Nervous System Activity: An Exploratory, Randomized Crossover Study
Source: J Clin Med. 2022 Sep 28;11(19):5738. doi: 10.3390/jcm11195738 (PMC9571939; doi:10.3390/jcm11195738)
Supplement: Supplementary file 1 [file jcm-11-05738-s001.zip › Supplementary_Materials_Tables S1 and S2.pdf]

Table S1. Summary statistics of mean heart rate and cardiovascular variability indices in the two baseline conditions and during the subsequent relaxing (Rel) and activating (Act) musical stimulation.

Variable	Rel Baseline	Rel Stimulation	Act Baseline	Act Stimulation
**HR, bpm**	72.8 (67.6, 82.0)	72.0 (64.1, 79.4)	72.8 (66.1, 80.3)	71.9 (66.1, 81.2)
**SDNN, ms**	44.3 (30.3, 48.3)	40.3 (32.6, 48.3)	41.7 (35.0, 54.1)	40.0 (34.2, 49.4)
**RMSSD, ms**	29.9 (21.4, 37.1)	31.4 (26.0, 42.5)	32.8 (28.1, 40.8)	27.9 (21.5, 38.9)
**pNN50, %**	7.8 (1.5, 16.7)	9.7 (4.5, 26.5)	8.8 (2.0, 18.1)	7.2 (1.5, 17.0)
**LF_{power}, ms^2**	487 (234, 778)	520 (395, 928)	369 (211, 1292)	405 (205, 657)
**LF_{NUr}, N.U.**	53.5 (36.1, 70.5)	54.0 (35.6, 68.1)	61.4 (37.4, 67.1)	59.0 (34.8, 68.4)
**HF_{power}, ms^2**	332 (272, 573)	504 (268, 710)	380 (225, 522)	391 (174, 544)
**LF/HF, A.U.**	1.15 (0.56, 2.39)	1.18 (0.55, 2.13)	1.59 (0.60, 2.04)	1.44 (0.53, 2.17)
**BRS, ms/mm Hg**	5.6 (3.5, 7.8)	6.4 (4.4, 8.6)	6.4 (3.9, 8.2)	5.7 (4.3, 7.2)

Summary statistics are expressed as median (Q1, Q3).
HR: mean heart rate; SDNN: standard deviation of normal-to-normal RR intervals; RMSSD: root mean square of successive squared differences of NN intervals; pNN50: proportion of interval differences of successive NN intervals greater than 50 ms; LF_{power}: low frequency power; HF_{power}: high frequency power; LF_{NU}: low frequency power in normalized units; LF/HF: LF_{power}/HF_{power}; A.U.: arbitrary units; BRS: baroreflex sensitivity.

Table S2. Summary statistics of the difference between the value of heart rate and cardiovascular variability indices during stimulation and that during the preceding baseline condition (stimulation effect), with the significance probability of the tests for the carryover and period effects, and for the difference between the effects of the two treatments.

Variable	Rel effect	Act effect	p Carryover	p Period	p Treatment
**HR, bpm**	-1.3 (-1.9, 0.0) ††	0.2 (-1.4, 0.9)	0.63	0.36	0.018
**SDNN, ms**	-2.5 (-3.1, 1.1)	-4.3 (-11.7, 0.6)†	1.0	0.82	0.31
**RMSSD, ms**	3.2(-0.2, 5.3)†	-1.6 (-6.5, -0.4)††	0.87	0.53	0.0002
**pNN50, %**	2.4 (-0.3, 5.1)†	-0.9 (-3.1, 0.4)	0.95	0.61	0.010
**LF_{power}, ms^2**	26 (-70, 179)*	-63 (-309, 49)*	0.72	0.47	0.015
**LF_{NU}, N.U.**	-1.7 (-10.9, 8.6)	0.8 (-9.7, 3.5)	0.97	0.88	0.89
**HF_{power}, ms^2**	61 (-37, 211)*	-16 (-109, 37)	0.69	0.78	0.05
**LF/HF, A.U.**	-0.02 (-0.68, 0.45)	0.02 (-0.68, 0.32)	0.40	0.85	0.72
**BRS, ms/mm Hg**	0.5 (-0.3, 2.3)†	-0.8 (-2.2, 0.6)	0.73	0.48	0.018

Summary statistics are expressed as median (Q1, Q3).

HR= mean heart rate; SDNN= standard deviation of normal-to-normal RR intervals; RMSSD= root mean square of successive squared differences of NN intervals; pNN50= proportion of interval differences of successive NN intervals greater than 50 ms; LF_{power}= low frequency power; HF_{power}= high frequency power; LF_{NU}= low frequency power in normalized units; LF/HF= LF_{power}/HF_{power}; A.U.= arbitrary units; BRS: baroreflex sensitivity. In some variables with non-normal distribution (RMSSD, LF_{power}, HF_{power}, LF/HF; see text), treatment effects were estimated using a non-parametric approach.

*) borderline non-significant (0.05< p <0.11); †) p< 0.05; ††) p< 0.005.
